# Detection of subclinical keratoconus using a novel combined tomographic and biomechanical model based on an automated decision tree

**DOI:** 10.1038/s41598-022-09160-6

**Published:** 2022-03-29

**Authors:** Peng Song, Shengwei Ren, Yu Liu, Pei Li, Qingyan Zeng

**Affiliations:** 1 Henan Provincial People’s Hospital, Henan Eye Hospital, Henan Eye Institute, People’s Hospital of Zhengzhou University, Zhengzhou, People’s Republic of China; 2 Aier Eye Hospital of Wuhan University, Wuhan, People’s Republic of China; 3 Wuhan Aier Hankou Eye Hospital, 328 Machang Road, Wuhan, People’s Republic of China; 4 Aier School of Ophthalmology and Optometry, Hubei University of Science and Technology, Xianning, People’s Republic of China; 5 Aier Cornea Institute, Beijing, People’s Republic of China; 6 Aier School of Ophthalmology, Central South University, Changsha, People’s Republic of China

**Keywords:** Corneal diseases, Predictive markers

## Abstract

Early detection of keratoconus is a crucial factor in monitoring its progression and making the decision to perform refractive surgery. The aim of this study was to use the decision tree technique in the classification and prediction of subclinical keratoconus (SKC). A total of 194 eyes (including 105 normal eyes and 89 with SKC) were included in the double-center retrospective study. Data were separately used for training and validation databases. The baseline variables were derived from tomography and biomechanical imaging. The decision tree models were generated using Chi-square automatic interaction detection (CHAID) and classification and regression tree (CART) algorithms based on the training database. The discriminating rules of the CART model selected metrics of the Belin/Ambrósio deviation (BAD-D), stiffness parameter at first applanation (SPA1), back eccentricity (Becc), and maximum pachymetric progression index in that order; On the other hand, the CHAID model selected BAD-D, deformation amplitude ratio, SPA1, and Becc. Further, the CART model allowed for discrimination between normal and SKC eyes with 92.2% accuracy, which was higher than that of the CHAID model (88.3%), BAD-D (82.0%), Corvis biomechanical index (CBI, 77.3%), and tomographic and biomechanical index (TBI, 78.1%). The discriminating performance of the CART model was validated with 92.4% accuracy, while the CHAID model was validated with 86.4% accuracy in the validation database. Thus, the CART model using tomography and biomechanical imaging was an excellent model for SKC screening and provided easy-to-understand discriminating rules.

## Introduction

Keratoconus is a progressive sight-threatening ectatic corneal disease that is one of the most common causes of corneal blindness in adolescents^1^. Due to the overlap in most parameters derived from topography and tomography imaging between subclinical keratoconus (SKC) and normal eyes (NE), identifying SKC remains one of the most challenging situations before making the decision to perform refractive surgery^2,3^. In the field of SKC screening, establishing combinations or models based on different algorithms has attracted increasing attention.

Currently, the strict criteria that define SKC are not well established. The inclusion criteria of subjects influence the outcomes of predictors’ discriminatory power in diagnosis studies. Previously, most studies only adopted topographic criteria to define SKC, which may have resulted in an overestimation of the model’s performance^4,5^. Hwang reported that a logistic regression model achieved high accuracy (AUC = 1.0) in detecting SKC with a normal topographic aspect^4^. Ambrósio et al. introduced a new combined index, the tomographic and biomechanical index (TBI)^6^, which was shown to exhibit a high AUC value of 0.985 in detecting SKC with a topography-based KISA% index^7^ of less than 60%. However, Steinberg et al.^8^ demonstrated that the discriminatory power of TBI decreased with an AUC of 0.825 in a validation research using the same inclusion and exclusion criteria as that in Ambrósio’s study^6^. The results of the aforementioned studies appear to be encouraging; however, when evaluating the performance of a model, the distribution of variables within the studied population must be seriously considered. Although the first detectable sign of keratoconus has not been definitively defined, previous studies highlight the importance of posterior surface abnormalities in SKC diagnosis^9,10^. Bae suggested that the back elevation difference (BED) must be included in the criteria of SKC in the future^3^. Therefore, future studies must be conducted with the aim of detecting SKC defined by stricter criteria that consider topography and posterior surface elevation.

Clinically, another troublesome aspect in SKC diagnosis is selecting the most useful variables from vast amounts of data and interpreting complex variables. In practice, the final decision before refractive surgery is often based on personal experience or proposed cutoffs of summary metrics in previous studies. Generally, an ideal model is easily interpretable, clinically credible, and statistically valid. A decision tree model represents a symbolic classification with “if–then rules,” which could provide easier interpretation than that of purely quantitative models, such as neural networks and logistic regression. Based on the decision tree algorithm, Smadja analyzed the metrics only from the GALILEI Dual-Scheimpflug Analyzer and reported an easy-to-understand discriminating rule and its desired accuracy for SKC screening^5^.

The aim of this study was to use the decision tree technique in the classification and prediction of subclinical keratoconus defined with normal topography and BED, considering the metrics proposed by tomography and biomechanical imaging.

## Materials and methods

This retrospective study was performed through a cooperative effort between Wuhan Aier Hankou Eye Hospital (Wuhan, China) and Henan Eye Hospital (Zhengzhou, China). Our study adhered to the tenets of the Declaration of Helsinki and was separately approved by the institutional review boards of the Wuhan Aier Hankou Eye Hospital and the Henan Eye Hospital. Written informed consent was obtained from each participant.

One hundred ninety-four participants were included in this double-center retrospective study. A total of 128 participants (70 healthy and 58 with very asymmetric ectasia) were enrolled from the Hankou Aier Eye Hospital (training database), and 66 participants (35 healthy and 31 with very asymmetric ectasia) were enrolled from the Henan Eye Hospital (validation database). All patients underwent comprehensive ophthalmic examinations, including a subjective analysis with slit-lamp biomicroscopy by an experienced anterior segment expert and objective examinations (i.e., Corvis ST and Pentacam HR examinations).

The objective definitions for normal and keratoconus were based on the well-established topographical keratoconus classification (TKC) index from topometric display and tomography imaging in the Pentacam system^3^. An eye was diagnosed as having keratoconus if it met with (1) a TKC index of greater than 0 and (2) at least one of the following signs: Vogt striae, Fleischer ring, Munson’s sign, and focal thinning. The diagnosis of keratoconus was only used to aid the definition of SKC and was not used for further analysis. The less affected fellow eye of an individual with very asymmetric ectasia was diagnosed as having SKC if it was characterized by (1) normal slit-lamp; (2) CDVA of 20/20 or better; (3) normal topographic aspect, a TKC index of 0^11^, the central mean keratometry value < 47.2D and I-S value < 1.40D^12^; and (4) BED < 12 μm^13^. The NE definition was characterized by the same criteria as that in SKC (both eyes of normal individuals met the criteria). All normal control patients underwent refractive surgery and had a two-year follow-up without any evidence of ectatic corneal changes. Only one eye was randomly selected for further analysis.

For all eyes included in the study, no contact lenses had been worn for at least four (rigid contact lenses) or two (soft contact lenses) weeks prior to examination; there was also no history of other eye diseases, no previous ocular surgery, and no use of topical medications other than artificial tears.

The techniques for Pentacam and Corvis ST analyses have been described previously^14,15^. The “QS” buttons from the Corvis ST and Pentacam HR read OK and the measured area of Belin/Ambrósio Enhanced Ectasia was no smaller than 8 mm (the measurement was included in the study). All cases from each hospital had tomographic data that were masked for re-evaluation by an anterior segment expert from the other center (SW and QY) to confirm recruitment.

Objectively, the variables analyzed included keratometry values, eccentricity, and astigmatism of the anterior and posterior surfaces; Kmax; the distance between any two points of the corneal apex, corneal thinnest corneal point, and pupil center point; maximum of pachymetric progression index (PPImax); Belin/Ambrósio deviation index (BAD-D); BED; back eccentricity (Becc); index of surface variance (ISV); index of vertical asymmetry (IVA); index of height asymmetry (IHA); index of height decentration (IHD); keratoconus index (KI); central keratoconus index (CKI); deformation amplitude ratio (DA-ratio); integrated radius (Integ-R); stiffness parameter at first applanation (SPA1); Corvis biomechanical index (CBI); and TBI.

### Description and tree-growing criteria for both the CHAID and CART decision trees

We used SPSS 22.0 software to generate decision trees based on Chi-square automatic interaction detection (CHAID) and classification and regression tree (CART) algorithms in the training database (the schematic theory of the discriminating rule based on a decision tree is presented in Supplementary Fig. 1). The tree-growing method of CHAID selects the independent variable that has the strongest interaction with the dependent variable. Further, Chi-square tests were used to determine node splitting and category merging. The significance level for node splitting in the CHAID model was a *P*-value less than 0.05. The CART method divides the data into segments that are as homogeneous as possible with respect to the dependent variable in the tree-growing procedure. In addition, the CART-growing method attempts to maximize within-node homogeneity, and the Gini coefficient was selected to measure impurity at the split. The minimum change in improvement was set as 0.0001. Furthermore, the pruning procedure was performed in the CART procedure to reduce overfitting.

### Growth limits

The maximum level of the tree depth was set to three for CHAID and five for CART. The minimum number of cases was set to ten for parent nodes and five for child nodes.

### Internal and external validation

In order to minimize the overfitting risk, a ten-fold cross-validation method was used for internal validation^16^. The external validation procedure was performed based on the validation database from Henan Eye Hospital. Further, statistical analyses were performed using SPSS 22.0 software and MedCalc software. The mean ± standard deviation (SD) and range were used to describe the data. All data were analyzed using the Kolmogorov–Smirnov normality test and Levene’s test for equal variances to select the appropriate method. The differences between the two groups were compared using the independent t-test or Mann–Whitney U test. The receiver operating characteristic (ROC) curve was used to test the performance of the studied metrics and decision tree models in different populations. Furthermore, DeLong’s test was used for the pairwise comparisons of area under the curve (AUCs). *P* < 0.05 (two-tailed) was considered statistically significant.

### Informed consent statement

Written informed consent was obtained from all individual participants included in the study.

## Results

**Figure 1:**
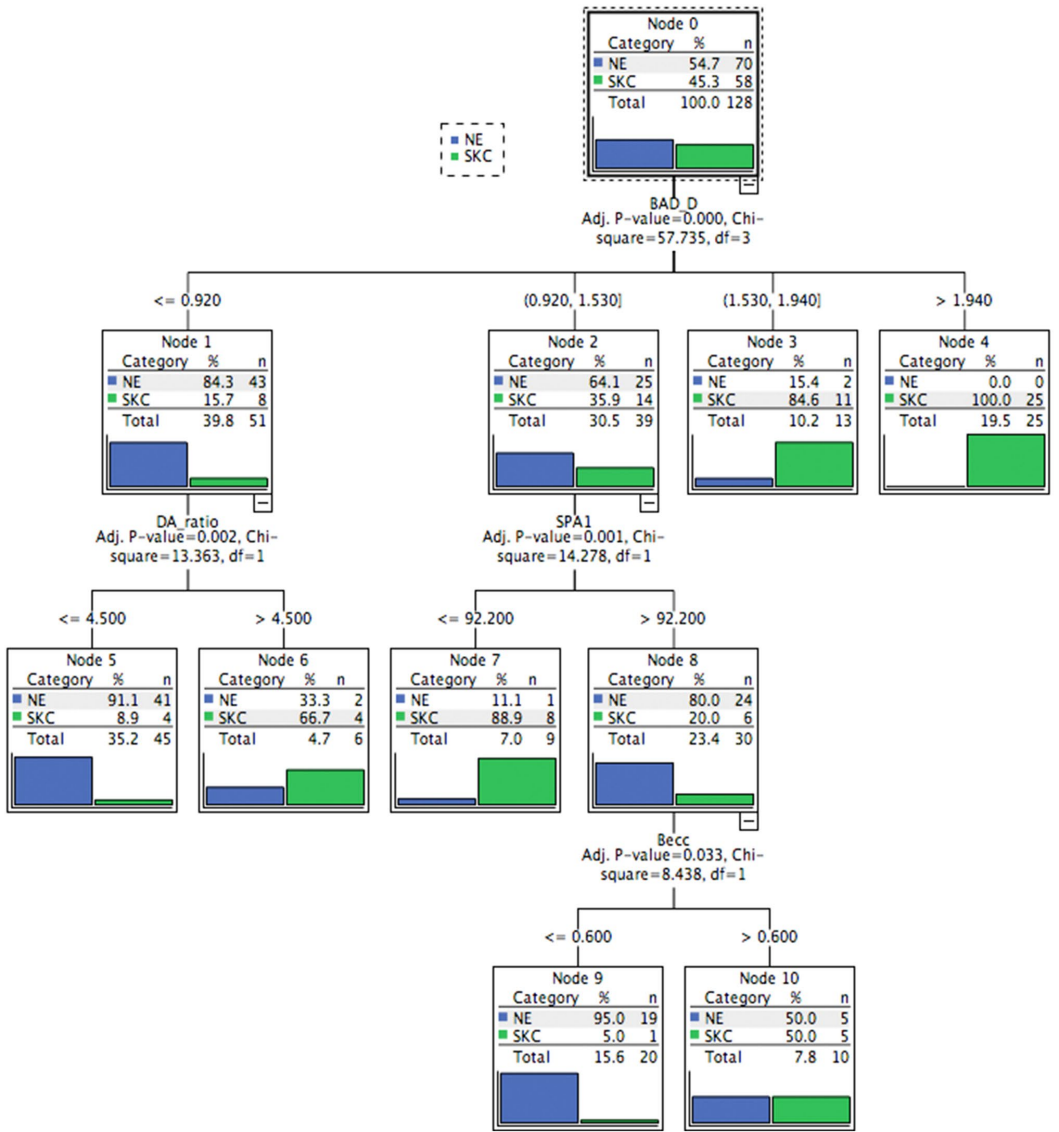
The CHAID model to discriminate between NE and SKC in the training database. This discriminating rule achieved 82.8% sensitivity and 92.9% specificity. When generating a split, the following cutoff values were obtained: BAD-D: 0.92, 1.53, and 1.94; DA-ratio, 4.5; SPA1, 92.2; and Becc, 0.6. Details of the splitting among various categories of eyes are specified in the boxes at each node. The category with the gray background was the targeted category. CHAID, Chi-square automatic interaction detection; NE, normal eye; SKC, subclinical keratoconus; BAD-D, Belin/Ambrósio deviation index; DA ratio, deformation amplitude ratio; SPA1, stiffness parameter at first applanation; Becc, back eccentricity.

**Figure 2:**
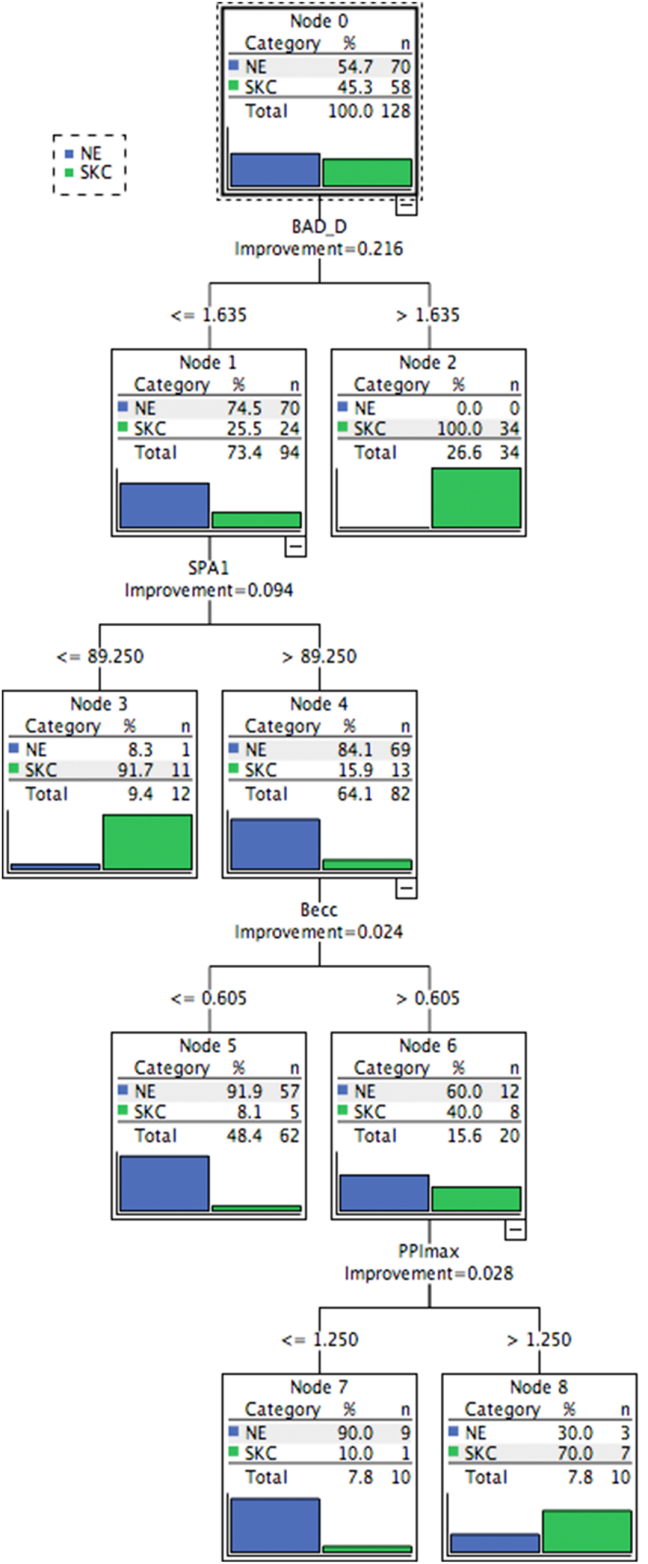
The CART model for discriminating between NE and SKC in the training database. This discriminating rule achieved 89.7% sensitivity and 94.3% specificity. When generating a split, the following cutoff values were obtained: BAD-D, 1.635; SPA1, 89.25; Becc, 0.605; and PPImax, 1.25. Details of the splitting among various categories of eyes are specified in the boxes at each node. The category with the gray background was the targeted category. CART, classification and regression tree; NE, normal eye; SKC, subclinical keratoconus; BAD-D, Belin/Ambrósio deviation index; SPA1, stiffness parameter at first applanation; Becc, back eccentricity; PPImax, maximum pachymetric progression index.

The predictive accuracies of the CHAID and CART models in the training database were 88.3% and 92.2%, respectively (Table 1). The CART model had a higher sensitivity (89.7%) than the CHAID model (82.8% sensitivity) (Table 1). The most important predictors in the CHAID model to discriminate NE and SKC included the BAD-D, DA-ratio, SPA1, and Becc (Fig. 1 and Table 2), whereas that in CART iNincluded the BAD-D, SPA1, Becc, and PPImax (Fig. 2 and Table 2). For both trees, the BAD-D was selected as the most discriminant variable to differentiate SKC and NE at the first split (with similar cutoff values of BAD-D: 1.53 in the CHAID model and 1.635 in the CART model). In addition, SPA1 was selected as the second-most discriminant variable for differentiating SKC from NE (cutoff values: 92.2 in the CHAID model and 89.25 in the CART model). The external validation procedure revealed that the CHAID model had 86.4% accuracy and that the CART model had 92.4% accuracy in the validation database (Table 1). The interpretation details of the discriminating rules of the CHAID and CART models are summarized in Table 2 and Figs. 1 and 2.

**Table 1:** The performance of CHAID and CART models to discriminate SKC from NE.

Model	Eye number (NE vs. SKC)	Accuracy (%)	Youden	Sensitivity (%)	Specificity (%)
CHAID	70 vs. 58	88.3	0.76	82.8	92.9
CART	70 vs. 58	92.2	0.84	89.7	94.3
CHAID*	35 vs. 31	86.4	0.73	90.3	82.9
CART*	35 vs. 31	92.4	0.85	90.3	94.3

CHAID, CHAID model based on training-database; CHAID, CART model based on training-database; CHAID*, the validation of CHAID; CART*, the validation of CART.

**Table 2:** Predictions of CHAID and CART for the outcome to discriminate NE and SKC in training database.

Node	Discriminating rules	Outcome	Probability (%)	Population
**CHAID model**				
4	If (BAD-D > 1.94)	SKC	100	25
3	If (1.53 < BAD-D ≤ 1.94)	SKC	84.6	11
5	If (BAD-D ≤ 0.92) and (DA-ratio ≤ 4.5)	NE	91.1	41
7	If (0.92 < BAD-D ≤ 1.53) and (SPA1 ≤ 92.2)	SKC	88.9	8
9	If (0.92 < BAD-D ≤ 1.53), (SPA1 > 92.2) and (Becc ≤ 0.60)	NE	95	19
**CART model**				
2	If (BAD-D > 1.635)	SKC	100	34
3	If (BAD-D ≤ 1.635) and (SPA1 ≤ 89.25)	SKC	91.7	11
5	If (BAD-D ≤ 1.635), (SPA1 > 89.25), and (Becc ≤ 0.605)	NE	91.9	57
7	If (BAD-D ≤ 1.635), (SPA1 > 89.25), (Becc > 0.605), and (PPImax ≤ 1.25)	NE	90	9
8	If (BAD-D ≤ 1.635), (SPA1 > 89.25), (Becc > 0.605) and (PPImax > 1.25)	SKC	70	7

*BAD-D* Belin/Ambrósio Enhanced Ectasia Display Index, *DA-ratio* deformation amplitude ratio, *SPA1* stiffness parameter at first applanation, *Becc* back eccentricity, *PPImax* maximum of pachymetric progression index.

**Figure 3:**
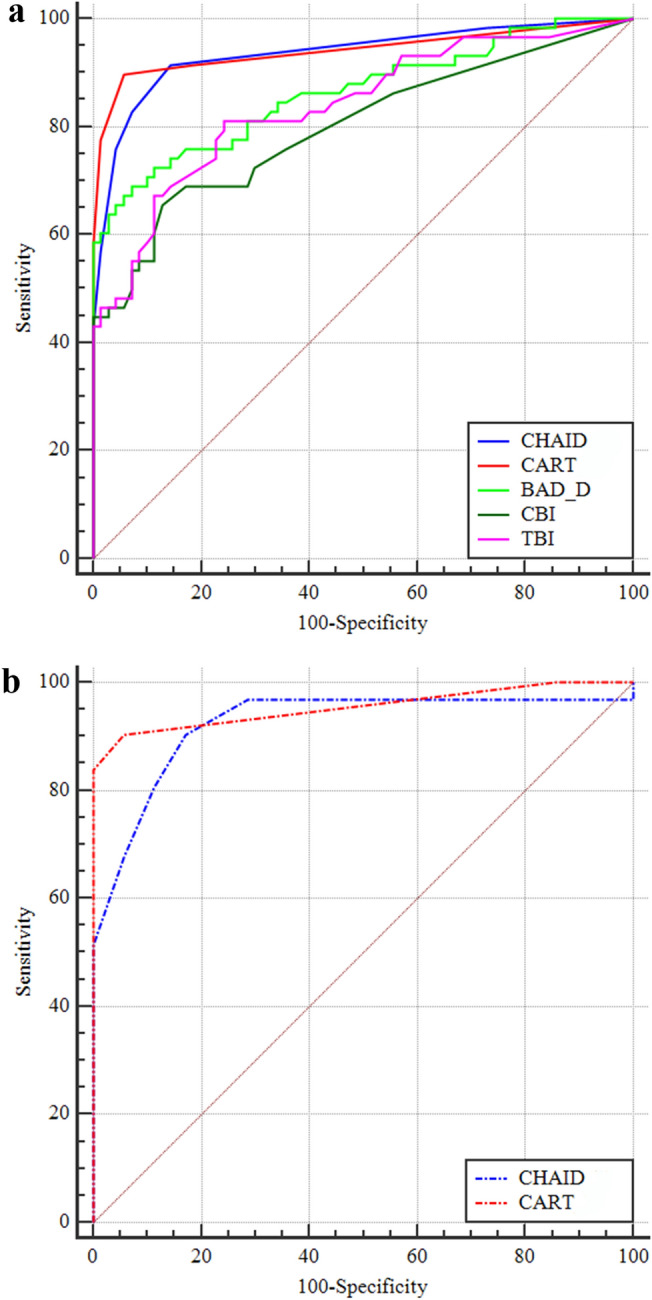
The ROC curves of the CHAID model, CART model, BAD-D, CBI, and TBI are presented in (**A**); the ROC curves of the two models in the validation database are presented in (**B**). ROC, receiver operating characteristic; BAD-D, Belin/Ambrósio deviation index; CBI, Corvis biomechanical index; TBI, tomographic and biomechanical index.

Descriptive statistics of the key studied variables are displayed in Table 3. Compared with those in NE eyes, the BAD-D, DA ratio, PPImax, Becc, CBI, and TBI were significantly higher, and the SPA1 was significantly lower in SKC eyes. The ROC results indicated that the BAD-D, SPA1, and PPImax had the highest AUC values of 0.86, followed by the DA-ratio and TBI (AUC values: 0.84 and 0.84, respectively). The AUCs of the CHAID and CART models were 0.94 and 0.94, respectively. In the validation database, the CHAID and CART models demonstrated similar AUC values (CHAID, 0.92; CART, 0.95) to those in the training database. DeLong’s test revealed that both the CHAID and CART models demonstrated significantly higher AUC values than each AUC of the individual BAD-D, CBI, and TBI (Table 4). The ROC curves of the CHAID model, CART model, BAD-D, CBI, and TBI are presented in Fig. 3A while the ROC curves of the CHAID and CART models of validation process are presented in Fig. 3B.

**Table 3:** Descriptive statistics of the key studied variables in the training-database.

Variable	NE	SKC	*P* value	AUC	95% CI	Cutoff	Accuracy (%)	Sensitivity (%)	Specificity (%)
BAD-D	0.79 ± 0.4	2.04 ± 1.00	< 0.001*	0.86	0.79–0.92	1.33	82.0	69.0	92.9
SPA1	111.9 (103.5, 121.6)	89.3 (81.0, 100.8)	< 0.001^a^	0.86	0.78–0.91	101.2	81.3	79.3	82.9
DA-ratio	4.3 (4.0, 4.4)	4.9 (4.5, 5.2)	< 0.001^a^	0.84	0.77–0.90	4.5	78.9	70.7	85.7
PPImax	1.24 (1.15, 1.32)	1.48 (1.34, 1.63)	< 0.001^a^	0.86	0.78–0.91	1.34	82.0	74.1	88.6
Becc	0.49 (0.43, 0.56)	0.60 (0.48, 0.65)	0.001^a^	0.68	0.59–0.76	0.54	67.2	63.8	70.0
CBI	0.01 (0, 0.04)	0.18 (0.02, 0.81)	< 0.001^a^	0.80	0.72–0.87	0.05	77.3	65.5	87.1
TBI	0.07 (0.01, 0.18)	0.35 (0.20, 1.00)	< 0.001^a^	0.84	0.76–0.90	0.17	78.1	81.0	75.7

*BAD-D* Belin/Ambrósio Enhanced Ectasia Display Index, *SPA1* stiffness parameter at first applanation, *DA-ratio* deformation amplitude ratio, *PPImax* maximum of pachymetric progression index, *Becc* eccentricity of back surface, *CBI* corvis biomechanical index, *TBI* combined tomography and biomechanical index;

*Independent t-test.

^a^Mann-Whitney U test.

**Table 4:** Delong’s test results of major predictors’ AUCs for NE vs. SKC.

	AUC difference	SE	95% CI	*P* value
BAD-D vs. CBI	0.06	0.04	− 0.02–0.15	0.158
BAD-D vs. TBI	0.02	0.03	− 0.04–0.09	0.420
TBI vs. CBI	0.04	0.05	− 0.06–0.13	0.422
CHAID vs. BAD-D	0.07	0.03	0.01–0.13	**0.014**
CHAID vs. CBI	0.14	0.04	0.05–0.22	**0.001**
CHAID vs. TBI	0.10	0.03	0.03–0.16	**0.004**
CART vs. BAD-D	0.08	0.03	0.02–0.14	**0.015**
CART vs. CBI	0.14	0.05	0.05–0.23	**0.002**
CART vs. TBI	0.10	0.03	0.04–0.17	**0.002**
CHAID vs. CART	0.01	0.02	− 0.03–0.05	0.781

*BAD-D* Belin/Ambrósio Enhanced Ectasia Display Index, *CBI* corvis biomechanical index, *TBI* combined tomography and biomechanical index, *CHAID* CHAID model based on training database, *CHAID* CART model based on training database.

Significant values are in bold.

## Discussion

In this study, we developed two models separately based on the CHAID and CART methods using the training database. The results revealed that the CART model had a higher discriminating accuracy of 92.2% (89.7% sensitivity and 94.3% specificity), whereas the CHAID model demonstrated 88.3% accuracy, with 82.8% sensitivity and 92.9% specificity. In this study, an independent validation database was included to evaluate the model for generalizability. In the external validation procedure, we found that the CART model had a similar predictive accuracy for SKC compared to its performance in the training database (Table 1). Furthermore, the external validation results revealed that the sensitivity of the CHAID model increased, whereas the specificity and overall predictive accuracy decreased compared with its performance in the training database. In terms of sensitivity and specificity, the performance of the CART model in the training and validation databases presented high consistency. Considering the severe outcomes of missed diagnosis of SKC, the CART model with higher predictive sensitivity and generalizability may be an optimal tool for ophthalmologists to detect SKC.

Based on different tree-growing methods, both CHAID and CART models selected BAD-D as the most discriminant variable to distinguish between the SKC and NE. Studies on SKC screening have confirmed the superior discriminatory power of the BAD-D over topographic variables and central thickness evaluation^17,18^. The cutoff values of the BAD-D used to discriminate SKC from NE were 1.6 in Shetty’s study^18^ and 1.09 in Steinberg’s study^8^, whereas the cutoff value of the individual BAD-D was 1.33 in our study. It must be noted that when selecting an optimal cutoff value, special attention must be given to the generalizability of the value within the studied population. In the present study, both the CHAID and CART models selected similar cutoff values (1.53 in the CHAID model and 1.635 in the CART model) of BAD-D to generate the first split, which were almost equal to the reference value of 1.6 between normal and suspected patients provided by the Pentacam system. Selecting a higher cutoff value of the BAD-D resulted in high screening specificity for SKC, while decreasing the false-positive rate in the first split of the decision trees. Taken together, these findings indicate that BAD-D must be considered most commonly in the clinical diagnostic rule of SKC.

Further, both models selected biomechanical variables as the second-most discriminant variable for differentiating SKC from NE (CHAID with DA-ratio and SPA1; CART with SPA1). Compared with the CHAID model with multiple branches, the CART model with a binary split nature incorporated a lower cutoff value of the SPA1, with 89.25 (SPA1 cutoff of 92.2 in the CHAID model). Generally, the CART tree-growing method attempts to maximize within-node homogeneity. Zhou reported that the SPA1 index, which reflects corneal stiffness, decreased and had a significant correlation with the thinnest corneal thickness in moderate-to-severe keratoconus but not in NE^19^. The DA-ratio reflects the corneal deformation level under specific air impulses; a weaker cornea is more prone to deformation. Previous studies have demonstrated that the individual DA-ratio and SPA1 can effectively discriminate keratoconus from NE but have worse performance in discriminating SKC from NE^20,21^. In our study, both the individual DA-ratio and SPA1 demonstrated the same AUC of 0.84 when comparing SKC and NE. However, the results in this study highlighted the importance of biomechanical variables in SKC diagnosis.

According to our findings, PPImax and Becc were not selected in the upper portion of either the CHAID or CART models. The PPImax represents the corneal thickness distribution from the thinnest point to the periphery, and the PPImax increases if the central cornea becomes thinner. This has been validated with higher accuracy for discriminating keratoconus from NE than that of the single pachymetric index^22^. We found that the PPImax had an AUC value of 0.86 for discriminating SKC from NE. Further, eccentricity is an important index for describing changes in corneal shape, and the normal value ranges from 0.4 to 0.6^23^. A previous study reported that eccentricity increases in keratoconus, even in its primary stages, with normal visual acuity of 1.0 and normal slit-lamp findings^24^. Although the AUC of individual Becc was low for differentiating SKC from NE in our study, collaboration with other variables in the decision trees strengthened its function in SKC screening.

When comparing the performance of the decision trees with other models, a major problem that arises is the nonconformity of studied populations among different studies. Smadja reported that a decision tree model based on Scheimpflug imaging parameters had a higher accuracy (96.4%) than that of our discriminating rules for detecting SKC^5^. In fact, stricter criteria (normal topography and BED) were used for SKC patient selection in our study. Atalay et al. developed a logistic regression model that combined metrics from tomography and ocular response analyzer imaging and reported that the model could detect SKC with 87.1% sensitivity and 91.4% specificity^25^. In their study, the inclusion criterion for SKC included BED as well; however, they included individuals who fell into the suspected category (BED ≤ 16 μm). Regardless of the differences in inclusion criteria, the predictive accuracy of decision tree models in our study was higher than that of the logistic-regression model in Atalay’s study.

Interestingly, using the same SKC criteria of (KISA% < 60%) and (I-S value < 1.45D), the AUCs of individual BAD-D, CBI, and TBI differed between Ambrósio’s study (AUCs for BAD-D,0.84; CBI, 0.82; TBI, 0.99)^6^ and Steinberg’s study (AUCs for BAD-D, 0.75; CBI, 0.79; TBI, 0.83)^8^ when comparing SKC and NE. According to the correlation between the KISA% index and BAD-D reported by Steinberg^26^, we robustly converted the BAD-D value of each individual in our study to KISA% and found that all included patients met the criteria of KISA% < 60%. Moreover, both Steinberg’s study^8^ and our study found that there were no significant differences between any two AUCs among those of the individual BAD-D, CBI, and TBI for differentiating SKC and NE. Furthermore, DeLong’s test revealed that both the CHAID and CART models had significantly higher AUCs than those of the individual BAD-D, CBI, and TBI. These findings confirmed that the performance of decision tree models in detecting SKC was superior to that of the summary combined metrics. However, to identify the superiority of the decision tree model, further studies based on different methodologies using our training and validation databases are required.

To investigate earlier changes in SKC, previous studies considered BAD-D analysis in the selection of SKC patients, which resulted in lower cutoffs with consuming sensitivity and specificity^8,12^. In our study, we did not consider BAD-D analysis when selecting SKC patients. First, the BAD-D analysis has been reported to have good performance in SKC screening, and we desired that the studied model include the efficient parameter BAD-D. Second, the strictest criteria with a normal BAD-D may result in a lower cutoff in the discriminating rules of decision tree and diminish the generalizability of the screening model in the entire population.

In conclusion, the decision tree models combined with metrics from tomography and biomechanical imaging showed excellent performance for discriminating SKC with normal topography and BED from NE, which highlighted the importance of overall analysis from broader features of the cornea. Moreover, the decision tree models generated visual and easy-to-understand discriminating rules to help ophthalmologists screen for SKC. Further, the BAD-D variable was the most critical determinant in classifying and identifying SKC, followed by SPA1.

## Supplementary Information

Supplementary Figure Legend.

Supplementary Figure 1.

## Data Availability

The data presented in this study are available on request from the corresponding author.
