# Supplementary material for: Detection of subclinical keratoconus using a novel combined tomographic and biomechanical model based on an automated decision tree
Source: Sci Rep. 2022 Mar 29;12:5316. doi: 10.1038/s41598-022-09160-6 (PMC8964676; doi:10.1038/s41598-022-09160-6)
Supplement: Supplementary file 1 — Supplementary Figure Legend. [file 41598_2022_9160_MOESM1_ESM.docx]

**Supplementary Figure 1 Legend**

Figure 1 Title: Schematic theory of the discriminating rule to differentiate between A and B based on a decision tree classification method

The primary split is based on discriminating variable 1 with a cutoff value of 1.645. The criterion of “if variable 1 is more than 1.645” targets the outcome of group B in node 2. Node 2 represents 38 individuals of group B who met this criterion, and no individuals of group A met this criterion. In other words, if individuals met this criterion, 100% of the individuals in group B were correctly labeled as “B”. Node 1 represents that the targeted category is A, and if individuals met “variable 1 is no more than 1.645,” the probability of being labeled “A” was 80.6%. In other words, 19.4% of individuals (12 individuals of group B) were incorrectly labeled as “A.” The second split was based on variable 2 with a cutoff value of 88.3. Node 3 represents that if the criteria of “variable 1 is no more than 1.645” and “variable 2 is no more than 88.3” were met, individuals of group B were labeled “B” with 100% probability. Node 4 represents 5 individuals of B who were incorrectly labeled “A.” In the particular decision tree, 50 individuals of group A were correctly classified, and 5 individuals of group B were misclassified (90% sensitivity and 100% specificity).
