# Supplementary figures and images for: Detection of subclinical keratoconus using a novel combined tomographic and biomechanical model based on an automated decision tree
Source: Sci Rep. 2022 Mar 29;12:5316. doi: 10.1038/s41598-022-09160-6 (PMC8964676; doi:10.1038/s41598-022-09160-6)

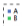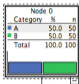

Variable 1  
Improvement = 0.306

$\leq 1.645$

$> 1.645$

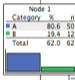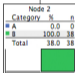

Variable 2  
Improvement = 0.104

$\leq 88.300$

$> 88.300$

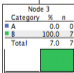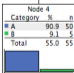

Supplement: Supplementary file 2 — Supplementary Figure 1. [file 41598_2022_9160_MOESM2_ESM.pdf]
